# Talking health: trusted health messengers and effective ways of delivering health messages for rural mothers in Southwest Ethiopia

**DOI:** 10.1186/s13690-019-0334-4

**Published:** 2019-02-21

**Authors:** Shifera Asfaw, Sudhakar Morankar, Muluemebet Abera, Abebe Mamo, Lakew Abebe, Nicole Bergen, Manisha A. Kulkarni, Ronald Labonté

**Affiliations:** 1 Department of Health, Behavior and society, Institute of health, Faculty of Public Health, Jimma University, P O Box: 378, Jimma, Ethiopia; 2 Department Population and Family health, Institute of health, Faculty of Public Health, Jimma University, P O Box: 378, Jimma, Ethiopia; 3 Faculty of Health Sciences, University of Ottawa, 600 Peter Morand Crescent, Ottawa, ON K1G 5Z3 Canada; 4 Faculty of Medicine, School of Epidemiology and PublicHealth, University of Ottawa, 600 Peter Morand Crescent, Ottawa, ON K1G 5Z3 Canada

**Keywords:** Talking health, Health messages, Health messengers, Ethiopia

## Abstract

**Background:**

Access to trusted health information has contribution to improve maternal and child health outcomes. However, limited research to date has explored the perceptions of communities regarding credible messenger and messaging in rural Ethiopia. Therefore, this study aimed to explore sources of trusted maternal health information and preferences for the mode of delivery of health information in Jimma Zone, Ethiopia; to inform safe motherhood implementation research project interventions.

**Method:**

An exploratory qualitative study was conducted in three districts of Jimma Zone, southwest of Ethiopia, in 2016. Twelve focus group discussions (FGDs) and twenty-four in-depth interviews (IDIs) were conducted among purposively selected study participants. FGDs and IDIs were conducted in the local language, and digital voice recordings were transcribed into English. All transcripts were read comprehensively, and a code book was developed to guide thematic analysis. Data were analyzed using Atlas.7.0.71 software.

**Result:**

Study Participants identified as Health Extension Workers (HEWs) and Health Development Army (HDA) as trusted health messengers. Regarding communication channels, participants primarily favored face-to-face/interpersonal communication channels, followed by mass media and traditional approaches like community conversation, traditional songs and role play.

In particular, the HEW home-to-home outreach program for health communication helped them to build trusting relationships with community members; However, HEWs felt the program was not adequately supported by the government.

**Conclusion:**

Health knowledge transfer success depends on trusted messengers and adaptable modes. The findings of this study suggest that HEWs are a credible messenger for health messaging in rural Ethiopia, especially when using an interpersonal message delivery approach. Therefore, government initiatives should strengthen the existing health extension packages by providing in-service and refresher training to health extension workers.

**Electronic supplementary material:**

The online version of this article (10.1186/s13690-019-0334-4) contains supplementary material, which is available to authorized users.

## Introduction

Communication interventions play an integral role in the delivery of health care and the promotion of healthy life styles, especially for mothers living in rural areas for whom access to health information regarding safe pregnancy and childbirth can contribute to lower maternal and neonatal morbidity and mortality. Information sharing has many purposes: initiating actions; making needs known; exchanging facts, ideas, attitudes and beliefs; facilitating a common understanding for better decision-making; and/or establishing and maintaining relations [1].

Health communication principles are used for a range of disease prevention efforts and health promotion strategies, including advocacy for health issues, marketing health plans and products, and community mobilization [2].

Communication scholars generally classify modes of health messaging into three main categories: interpersonal, mass media and traditional [2–4]. Interpersonal approaches include exchanges between various types of health care providers’ (e.g. doctors, nurses and community health workers), and those outside the health profession (e.g. family, friends, and informal community groups) [3]. This approach is preferred for the transmit of information and teaching of skills to encourage desired behavior changes [5].

Mass media communication channels transmit information for target audiences through television, radio, and written materials such as posters, books, leaflets, magazines, and newspapers. They are primarily focused on reaching large segments of population [6].

On the other hand, communities may use their own traditional approaches to message delivery, including songs, drama, storytelling, role-play, proverbs, community meetings and poems. Previous studies have shown that traditional modes of communication and knowledge transfer are preferable for poor and rural populations since they usually do not require advanced technology, they are typically very interesting and emotive, and they are easily understood and accepted by the community [4, 5].

Community use of health information varies with situational and personal differences. Understandably, individuals who consult multiple sources of information are more likely to make an informed decision about health than individual who rely on a single source [7]. A recent study on the dissemination of health information in Africa supports the utilization of multiple source and channels available in the local community [4]. However, explorations of which and why particular source /messengers and channel are preferable for local audiences are lacking.

In Ethiopia, trusted health messengers and preferred channels have not been well explored and documented, particularly in the area of maternal health. Assessing trusted messengers and messaging is important for effective health knowledge transfer and behavior change. A better-informed household may make an informed decision on seeking maternal health services during different stages of maternity (i.e. during pregnancy, at delivery, or following childbirth). Having access to health information also reduces delays to get appropriate maternal health care services. Therefore, this study explored credible maternal health messengers and preferred modes of message delivery approaches as perceived by rural mothers in Jimma Zone.

The study is a part of an ‘innovative for maternal and child health Africa project (IMCHA) entitled: ‘*an implementation study of intervention to promoting safe motherhood in Jimma zone, Ethiopia*’. The IMCHA project used a randomized cluster intervention design, in which two interventions associated with delays in women seeking prenatal care or health facility delivery were trialed: fully operational Maternal Waiting Areas (to promote safe delivery in a health facility), and new ‘information, education, and communication’ (IEC) programs. In this article we report on findings from a qualitative study of community-level communication preferences for IEC messaging and delivery, with the goal of informing the IEC intervention aspect of the research. Our findings should also assist decision makers and other stakeholders who are working to improve maternal and child health outcomes in the design of health communication strategies that align with community interests and preferences.

### Research questions

This research aimed to answer the following questions:

1. Who are trusted health messengers in the community?
2. What are the community’s preferred communication channels?
3. Why do people trust a source of information over another?

## Methods

### Study setting

The study was conducted in Jimma Zone, located in the southwest region of Ethiopia. Jimma town is 346 km from Addis Ababa, the capital of Ethiopia. The Zone is known for its production of “*Coffee Arabica*” which is the back bone of the country’s economy. Jimma town has located a latitude and longitude of 7°40′N 36°50′E.

Jimma Zone has 21 districts. Among these, three districts were purposively selected for this project (Gomma, Seka Chekorsa and Kersa) by considering of high population size and low health service utilization. The number of health centers, health posts, HEWs and Health development army leaders in the selected three districts were 28, 110,231 and 3384 respectively [8]. The study was conducted in May and June 2016.

### Research approach

This research used an exploratory qualitative case study to better understand participants’ understanding of trusted maternal health information sources and their preferred communication channels. For purposes of this study, trusted messengers were defined as persons regarded by community members as credible sources of health information for pregnant women in making informed decisions regarding health maintenance and health seeking behavior. Preferred communication channels described the different means or venues in which community members liked to receive IEC messaging. These preferences were assessed by in-depth interviews (IDI) and focus group discussion (FGDs). The FGDs and IDIs were considered to create an appropriate context for the researchers to explore community member feelings, perceptions, and understanding of the maternal/child health (MCH) topics that would form part of the IEC intervention.

### Sample size and technique

Data collection involved IDI and FGDs. Six individual depth-interview were conducted for each of the following stakeholder groups: religious leaders, health extension workers (HEWs), and members of the Women Development Army (WDA) and Male Development Army (MDA)^1^(total *N* = 24). Six FGDs were held among female community members and another six with male community members (see Appendices 1–6 for a detailed sampling overview).

### Data collection and analysis

Researchers developed IDI and FGD guides based on a review of literatures in the areas of health communication, message development, and message delivery approaches, with a focus on improving maternal and child health outcomes associated with pregnancy and childbirth [9–11]. These instruments were tailored to explore the different experiences, perceptions, and roles of each group of study participants (HEW, WDA, MDA, religious leaders, male community members, and female community members). Each guide contains questions on basic socio-demographic variables like age, sex, educational status and role in the community, as well as questions and probes on existing and preferred IEC programs related to improving MCH outcomes in rural contexts.

Written and/or oral consent was obtained from all participants. IDIs and FGDs were conducted in convenient, quiet and private locations in order to ensure confidentiality. Data were collected using digital audio recorders and field note memos were taken to document non-verbal or other behaviors observed during the data collection. Audiotapes and notes were transcribed following data collection. *Atlas.ti 7.0.71* software was used for data analysis.

A rough outline of thematic categories was drafted based on the themes that were specified in the FGDs and IDI guidelines. This outline was further developed into a preliminary code guide. Next, all FGD and IDI transcripts were read multiple times by the research team to produce a final code guide. To enhance inter-coder reliability, the coders independently applied the guide to all transcripts; discrepancies were reviewed and resolved. This exercise ensured that the coders had a common understanding of the code guide and its application. Exemplary quotations for selected codes were generated in *Atlas.ti* software using the Code Manager/Output feature. The summaries presented below reflect both widely-expressed ideas, as well as novel ideas that were mentioned less frequently.

### Maintaining research trustworthiness

To promote credibility, data collection tools were pretested in similar contexts to maximize the validity of the tool. The IDI and FGD questions were open-ended and participants were encouraged to discuss the questions in an uninhibited manner while being guided to remain focused on the topic of interest. To promote transferability, appropriate probes were used to obtain detailed information on responses. Detailed field notes were taken, and all interviews were digitally recorded (thick description).

To address confirmability, this study employed reflectivity and bracketing methods. These methods helped to minimize respondent bias and the risk of reactivity whereby participants could deny information due to the presence of data collectors and researcher, while “bracketing out” daily debriefing sessions provided an opportunity for the data collectors and researchers to explore how their own preconceived ideas might be affecting the study, and to increase their reflexivity in later interpreting the findings.

To promote dependability, all data collectors were bilingual (fluent in both English and the local languages) and trained at the post-graduate level. They also had prior experience in qualitative data collection, and had participated in an intensive, week-long training program prior to undertaking field research. Interviews were conducted until data saturation was reached, within the limits imposed by geography and time-frame for the study. The duration of the interviews and discussions ranged from 45 to 90 min (prolonged engagement).

## Results

### Socio-demographic characteristics of the respondents

The mean age of participants who were participated in all in-depth interviews (Male Development army (MDA), women development army (WDA), and HEW groups) were 46.5, 37.7, and 25.8 years, respectively. The mean age of the religious leaders was 52.5 years. The maximum service years as a religious leader was 45 and minimum of below 15 years (See annex 1–6). Regarding FGD participants, in each of three districts a total of two kebeles were selected to conduct FGD among male and female community members with nine to twelve participants (see additional file 1). The age of IDI participants from different groups with their respective district is presented below in Table 1.

**Table 1:** Age of the IDI participants in Jimma zone south west of Ethiopia, 2016

Study site		In-depth interview participants			
		MDA	WDA	Religious Leaders	HEWs
District	Kebele	Age	Age	Age	Age
Kersa	Baallto	48	28	–	30
	Kitimbile	50	28	39	24
Seka Chekoresa	Hula Huke	46	42	65	23
	Buyo Kechema	45	35	70	25
Gomma	Keso Hito	35	53	–	25
	Kilole	55	40	36	28

(−) = missed to record

### Summary of findings by thematic categories

Most of the participants consider HEWs to be credible health messengers due to the closeness of the source and their position in the community as a trained health worker. Family and community members and other health workers were also mentioned as secondary health messengers. Sometimes, guests from outside of the community were seen as acceptable messengers.

### Theme- 1: Preferred messengers

#### Category-1: Health extension workers

Study participants reported that HEWs were generally the most credible source of health information. They provide education and knowledge to keep the community healthy.

*“HEW give us awareness by going house to house to reach each household. They also give information when mothers go to their office. In this way mothers are advised to access health care and to modify their lifestyles”.*

**(Female community member, Kersa district, age 25).**

Most participants favoured HEWs over other sources of health information. Participants spoke of HEWs as ‘heroes’ or ‘angels’ for the community since they perceived that HEWs are near to them to respond to immediate needs. They teach the community about the importance of a healthy lifestyle, nutrition and hygiene, and advise the community to use health facilities. At the same time, they work to end unhealthy traditional practices and encourage husbands to support their wives.

“*We prefer health extension workers. We have many reasons: we meet them physically and answer our questions empathetically. Sometimes health professionals from district health office came and teach us about health and healthy life like utilization of maternal and child health cares”.*

**(Female community member, Gomma district, age 27**)

*“…For instance, regarding to sanitation practice, what health extension workers advised us has been more advantageous and important than media. The information helps us to build and use latrines”.*

**(Male community member, Kersa district, age 28)**

### Category-2: Community members and other government bodies

Information promoting maternal and child health also came from other community members, notably religious leaders and members of the MDA and WDA.

*“…. waiting for health extension workers may not always work; the community should educate one another at “garees” [an arrangement within a village which contains at least thirty households and is led by one leader] level. Then, the garee leader will educate the “shanee” (a group of five households in the garee structure). Religious leaders also teach the community”.*

**(Male community member, Seka Chekorsa district, age 55)**

Some participants preferred to get health information from government bodies other than HEWs or district health offices, or from other officials such as Kebele leaders.

*“I prefer the advice from government bodies; I believe and accept them since the government sent for me. I already started applying the information at home for example, my children are always washing their hand”.*

**(Female community member, Seka Chekorsa district, age 55)**

### Theme-2: Preferred messaging

#### Category-1: Interpersonal approach

Channels of health information preferences were assessed by asking participants about different sources, and how they preferred to receive health information. Participants shared their opinions on preferred message delivery approach and the reason behind their preferences.

Participants indicated that face-to-face communication is a preferred mode for receiving health information and for gaining skills to adopt healthy behaviors. They noted certain advantages related to feelings of trust and engagement.

*“Teaching community through face to face conversation is better than media. Most people didn’t trust what they heard from television or radio. Therefore, it better to teach through face-to-face communication to make lively”.*

**(Male community member, Kersa district, age 29)**

### Category-2: Mass media approach

#### Radio, Television and printed materials

Participants mentioned that radio, television and written materials are the main means of mass communication and noted advantages and disadvantages of these sources. In some cases, radio was the preferred media.

*“Radio is preferable to reach everyone in the locality. The HEWs can’t cover everywhere as radio do. The appropriate timing for listening radio is after 2:00 pm since all household members are free at this time”.*

***(*** **Female community member, Kersa district, age 26).**

Although some people preferred getting information through television “because we can see every demonstration”, as one Kersa district community member noted, others from the same district pointed out that “only few households possess televisions”. A community member from Kersa district justifies her reason as follow.

*“I preferred television because we can see every demonstration and radio could be my second preferences”.*

**(Female community member, Kersa district, age 25)**

Participants said that written materials such as booklets, posters, and pamphlets were useful channels for accessing health information. For instance, one female community member outlined the type of information obtained through written materials.

*“I favored GALMEE [family folders] and secondly KARTA [information booklets] because we can keep them in our homes. For instance, we have one blue and one yellow colored folder in our home. It contains health information about pregnancy, personal hygiene and environmental sanitation. It has writing words and photographs. That is why I prefer this approach”.*

**(WDA, Seka Chekoresa district, age 26)**

A HEW spoke about the advantages of using written material to supplement teaching activity:

*“If they are unable to read, they can see pictures from posters. Many individuals also able to read Amharic since they learned during previous government”.*

**(HEW, Seka Chekoresa district, age 23)**

A religious leader from the same district also spoke about the importance of written materials as communication channels to the local community.

*“I prefer leaflets, since it can be stay long at home. Leaflets can be read by school children to their family. It is more understandable and has more details”.*

**(Religious leader, Seka Chekorsa district, age 65)**

### Category-3: Traditional ways of messaging approach

Information delivery channels included more traditional modes, such as community meetings, community conversations, traditional songs, dramas, role play and informal training sessions. Other participants mentioned that they were interested in receiving education from guests or higher-level authorities, while others talked about having continuous and planned health education sessions that focus on local health problems. The opportunity to have question-and-answer periods and to use mixed methods were appealing to the community. One male community member commented about the efficiency of holding community meetings.

*“Look, if we provide information through meeting, like a group of 300 people per village, then, these 300 peoples teach their family members. At last, we can educate the whole village. Therefore, if we want to be successful, meeting is the best option than other modes”.*

**(Male community member, Gomma district, age 42).**

Another male community member spoke about his preference to learn through dramas.

*“I saw in one drama that; one person was drinking “TEJ” [a local alcoholic drink]. His friend came and sat beside him and encouraged him to drink more “TEJ”. Then the person said, “No”, I had enough; my stomach is full. It is time to buy something for my children. Then he bought eggs and went to home. He then made porridge with egg and started to feed his children. This is very good education to learn from such dramas”.*

**(Male community member, Gomma district, age 45)**

Another participant suggested the importance of being trained by those people who attended TOT (training of trainers) courses outside of the community.

*“It is good if someone takes some training at district level or somewhere else, he/she can be back and give some awareness to the community”.*

**(MDA, Kersa district, age 48)**

Another participant justified the value of training over other methods.

*“If we take the leaflet, we kept in our wallet, the drama also effective only for children. So, the training is effective for adult and mothers”.*

**(Male community member, Seka Chekorsa District, age 27)**

HEWs also saw value in using traditional methods of communication. For example, a HEW from Kersa district saw value in holding community conversations.

*“…. community conversation is very important for intended community interaction and to promote health issue. It’s very helpful to communicate health information”.*

**(HEW, Kersa District, age 30)**

## Discussion

This study explored various sources of health information and identified trusted and appropriate health communication channels in rural communities in Jimma Zone, southwest of Ethiopia. The HEWs and health care providers, along with others (WDA, MDA, family members, religious leaders, and people from outside the community) were mentioned as the principal sources of health information. Almost all participants agreed that health information from HEWs was trusted and relevant to decision-making regarding health. This finding is consistent with an earlier study conducted in Nigeria where community health workers were identified as the most available and reliable sources of health information [9]. Another study also found that maternal-related health information seeking is seen as trusted and ‘unbiased’ if it comes from health workers [10]. This may explain why health workers are among the most respected members of the social group on whom the community relied.

Health extension workers in Ethiopian health system are similar to such health workers in many other low- and middle-income countries [12]. They mandated to address maternal and child health service utilization and basic communicable disease prevention and control strategies. Participants in our study also spoke of HEWs’ effectiveness in mobilizing the community towards promoting health and wellbeing, consistent with findings from an earlier study [13]. This effectiveness is likely attributable to participants’ emphasis on how HEWs made a home to home visits on a regular basis, creating a closeness and sense of trust between them; unsurprisingly, our study found that HEWs were regarded as the nearest and most empathic health information source for the community. Importantly, researchers in the area of communication study further find that women are more empowered to make informed choices and to report satisfaction in health promotion and disease prevention activities when encouragement to do so is provided by their preferred source of health information [14], in our study this being primarily HEWs.

The study, however, also found that credible health information was provided by other community members, including religious leaders, and WDA and MDA members, and from existing government structures like kebele and district officials.

In Ethiopia, sub district and district leaders have significant political influence in the community.

With respect to suitable community communication channels, most study participants considered interpersonal (face-to-face) communication to be the most important, followed by mass media and traditional communications channels. Participants preferred face-to-face interpersonal communication channels due to its personable nature, trustworthiness, and the opportunity for dialogue (feedback) between the people providing or receiving health information. Other study conducted among Somalia women in United Kingdom find similar reasoning for preference of interpersonal communication over other channels [15].

Moreover, most of the participants in our study reported that interpersonal communication approach was preferable because it is affordable and adaptable for rural settings, similar to findings from other study [11].

Communication scholars also consider interpersonal communication channels to be the standard by which other channels (such as mass media) should be measured, since interpersonal communication utilizes all the senses and enables communication partners to incorporate immediate feedback [16].

Another reason for the preference of interpersonal communication found in our study may be related to most rural mothers being illiterate with low health literacy levels, potentially creating difficulties in their understanding of complex messaging from mass media and/or traditional sources.

Participants were more varied in their opinions about mass media sources for receiving health message, such as television, radio and written materials. Radio was mentioned as a good medium because it could reach a large segment of the population, most households had radios, and it did not require a high literacy level to operate. This finding is consistent with a previous study conducted in Nigeria where most mothers preferred to receive health information through radio [17]. In contrast, another study done in Pakistan revealed that a majority of mothers preferred to get health information through television rather than by radio [18]. Differences in preferred mass media channels likely reflect the availability of and accessibility to different media, as one of our study participants noting specifically that few community members had televisions.

Few study participants preferred to get health information through written material, such as folders and booklets that are still frequently used in health education campaigns. This option was likely less popular than other communication channels owing to the high illiteracy rates in the communities, and because few have a habit of reading.

The importance of promoting literacy and the improvement of reading habits cannot be overstated in their importance to overall human development, especially for mothers [19]. This implies that before considering written material as a communication channel for health information in places where literacy is low, broader initiatives to improve access to education are warranted. Health communication practitioners should consider the characteristics and abilities of the target audiences, especially their literacy status and media habits. To an extent, then, written forms of health information may be seen primarily as tools to improve literacy, and only secondarily as one form of imparting important health knowledge.

That health communication, regardless of media used, often serve other purposes was apparent in participants’ appreciation for traditional communication channels, such as dramas, role play and community meetings. Such activities, apart from being useful for sharing maternal health issues and communicating new health knowledge, were also popular because they were lively and entertaining, as well as educational.

A systematic review on the effectiveness of behavior change communication for improving maternal health outcome also revealed that drama, meetings, and community conversation were important communication channels for rural mothers as well as for rural men since both share similar household asset except the gender role [20].

### Limitation and strength of the study

This study has a limitation related to design. The finding of this study may not be applied for general population. The context of the study also may not fully applicable to other setting. Some of the participant may be exaggerate the activity of health extension worker (one of the stake holders) due to their intimacy with the local community. However, the Authors tried to keep the trustworthiness of the data using thick discerption, prolonged engagement, memo making and by triangulating two qualitative data collection methods (FGD and IDI).

## Conclusion

This study sought to identify what community members regarded as credible health messengers and preferred mode of message delivery approach in rural Ethiopia. Our main findings suggest that, most participants prefer to get health information from HEWs through interpersonal interactions. Participants believed that HEWs were the nearest guardians of their health and were widely considered to be community champions. Based on our findings, governments should support and strengthen the role of HEWs at the community level. The HEWs should be motivated through provision of training and educational opportunity.

## Additional file

Additional file 1: Detail sociodemographic characteristics of FGD and IDI participants. (DOCX 14 kb)

## Abbreviation

FGD: Focus Group Discussion
HEW: Health Extension Worker
IDI: In-Depth Interview
IEC: Information Education and Communication
IMCHA: Innovative Maternal and Child Health Africa
MDA: Male Development Army
RL: Religious Leader
WDA: Women Development Army

## Footnotes

1
*Health Development Armies are comprised of two persons -- one female (for maternal/child health components) and one male or female, but generally male (for environment and sanitation components). Health Development Army members are appointed from a cluster of five households (the tokko-shanee, meaning 1:5) and are responsible for ensuring that the five households are aware of, and follow, the health practices associated with the Ethiopian Health Extension Program.*

## References

[1] United States (1998). Office of Public Health, United States Office of Disease Prevention, Health Promotion Healthy people 2010 objectives: Draft for public comment US Government Printing Office.

[2] Thomas RK. Health communication: Springer Science & Business Media; 2006.

[3] Johnson JD, Meischke H (1993). A comprehensive model of cancer-related information seeking applied to magazines. Hum Commun Res.

[4] Mills ME, Davidson R (2002). Cancer patients’ sources of information: use and quality issues. Psycho-oncology: journal of the psychological, social and behavioral dimensions of. Cancer.

[5] Anasi SN (2012). Access to and dissemination of health information in Africa: the patient and the public. J Hosp Librariansh.

[6] Parrott R (2004). Emphasizing “communication” in health communication. J Commun.

[7] De Pietro R, Clark NM (1984). A sense-making approach to understanding adolescents’ selection of health information sources. Health Educ Q.

[8] Jimma zone health office (2018). Health service coverage in Jimma zone.

[9] Onuoha UD, Amuda AA. Information seeking behaviour of pregnant women in selected hospitals of Ibadan metropolis. J Inf Knowl Manag. 2013;4(1).

[10] Garnweidner LM, Pettersen KS, Mosdøl A (2013). Experiences with nutrition-related information during antenatal care of pregnant women of different ethnic backgrounds residing in the area of Oslo, Norway. Midwifery.

[11] Hossain A, Islam S (2012). Information needs of rural women: a study of three villages of Bangladesh.

[12] Labonte R, Sanders D, Packer C, Schaay N (2014). Is the Alma Ata vision of comprehensive primary health care viable? Findings from an international project. Glob Health Action.

[13] Glenton C, Javadi D (2013). Community health worker roles and tasks.

[14] Hibbard JH, Greenlick M, Jimison H, Kunkel L, Tusler M (1999). Prevalence and predictors of the use of self-care resources grading system. Eval Health Prof.

[15] Davies MM, Bath PA (2002). Interpersonal sources of health and maternity information for Somali women living in the UK: information seeking and evaluation. J Doc.

[16] Kiousis S (2002). Interactivity: a concept explication. New Media Soc.

[17] Ogunmodede TA, Sarah A, Olusegun S. Health information need and information sources of pregnant women in Ogbomoso Metropolis, Oyo State, Nigeria

[18] Mustafa R, Afreen U, Hashmi HA (2008). Contraceptive knowledge, attitude and practice among rural women. J Coll Physicians Surg Pak.

[19] Dent VF (2007). Local economic development in Uganda and the connection to rural community libraries and literacy. New Libr World.

[20] Nair SN, Darak S, Parsekar SS, Menon S, Parchure R, Vijayamma R, Darak T, Nelson H. Effectiveness of behaviour Change Communication (BCC) interventions in delivering health messages on antenatal care for improving Maternal and Child Health (MCH) indicators in a limited literacy setting: an evidence summary of systematic reviews.

