# Supplementary material for: Talking health: trusted health messengers and effective ways of delivering health messages for rural mothers in Southwest Ethiopia
Source: Arch Public Health. 2019 Feb 21;77:8. doi: 10.1186/s13690-019-0334-4 (PMC6383212; doi:10.1186/s13690-019-0334-4)
Supplement: Supplementary file 1 — Detail sociodemographic characteristics of FGD and IDI participants. (DOCX 14 kb) [file 13690_2019_334_MOESM1_ESM.docx]

FGD and in-depth interview participants

**Annexes**

Appendix 1. Focus group discussion: Female community members

| **District** | **Kebele** | **Number of participants** |
| --- | --- | --- |
| Kersa | Baallto | 11 |
|  | Kitimbile | 9 |
| Seka Chekoresa | Hula Huke seqa | 11 |
|  | Buyo Kechema | 12 |
| Gomma | Keso Hito /Gomma | 11 |
|  | Kilole | 11 |

Appendix 2. Focus group discussion: Male community members

| **District** | **Kebele** | **Number of participants** |
| --- | --- | --- |
| Kersa | Baallto | 12 |
|  | Kitimbile | 9 |
| Seka Chekoresa | Hula Huke | 7 |
|  | Buyo Kechema | 6 |
| Gomma | Keso Hito | 7 |
|  | Kilole | 9 |

Appendix 3. Participant demographic information: Health Extension Workers

| **District** | **Kebele** | **Age** | **Sex** | **Years in role** |
| --- | --- | --- | --- | --- |
| Kersa | Baallto | 30 | Female | 10 |
|  | Kitimbile | 24 | Female | [missing] |
| Seka Chekoresa | Hula Huke | 23 | Female | 7 |
|  | Buyo Kechema | 25 | Female | 8 |
| Gomma | Keso Hito | 25 | Female | 4 |
|  | Kilole | 28 | Female | 10 |

Appendix 4. Participant demographic information: WDA

| **District** | **Kebele** | **Age** | **Sex** | **Years in role** |
| --- | --- | --- | --- | --- |
| Kersa | Baallto | 28 | Female | 4 |
|  | Kitimbile | 28 | Female | [error] |
| Seka Chekoresa | Hula Huke | 42 | Female | 10 |
|  | Buyo Kechema | 35 | Female | [missing] |
| Gomma | Keso Hito | 53 | Female | 10 |
|  | Kilole | 40 | Female | 2 |

Appendix 5. Participant demographic information : MDA

| **District** | **Kebele** | **Age** | **Sex** | **Years in role** |
| --- | --- | --- | --- | --- |
| Kersa | Baallto | 48 | Male | >15 years |
|  | Kitimbile | 50 | Male | 30 |
| Seka Chekoresa | Hula Huke | 46 | Male | 26 |
|  | Buyo Kechema | 45 | Male | [error] |
| Gomma | Keso Hito | 35 | Male | 8 |
|  | Kilole | 55 | Male | 28 |

Appendix 6. Participant demographic information : Religions Leaders

| **District** | **Kebele** | **Age** | **Sex** | **Religion** | **Years in role** |
| --- | --- | --- | --- | --- | --- |
| Kersa | Baallto | [missing] | Male | [missing] | >15 |
|  | Kitimbile | 39 | Male | Muslim | 18 |
| Seka Chekoresa | Hula Huke | 65 | Male | [missing] | [missing] |
|  | Buyo Kechema | 70 | Male | Muslim | 45 |
| Gomma | Keso Hito | [missing] | Male | [missing] | [missing] |
|  | Kilole | 36 | Male | [missing] | 18 |
